# Determining the optimal cutoff value of prostate-specific antigen density for diagnosing prostate cancer in 50–80-year-old patients undergoing prostate biopsy with PSA levels of 4–10 ng/mL

**DOI:** 10.1097/MS9.0000000000005782

**Published:** 2026-10-27

**Authors:** Niusha Jamshidi, Abazar Akbarzadehpasha, Ali Bijani, Mohammad-Bagher Rajabalian, Seyed Mohammadhasan Hosseini, Emadodin Moudi

**Affiliations:** a Student Research Committee, Babol University of Medical Sciences, Babol, Iran; b Clinical Research Development Center, Shahid Beheshti Hospital, Babol University of Medical Sciences, Babol, Iran; c Social Determinant of Health Research Center, Babol University of Medical Sciences, Babol, Iran

**Keywords:** gray zone PSA, prostate biopsy, prostate cancer, prostate-specific antigen density, total PSA

## Abstract

**Background:**

Prostate-specific antigen (PSA) has weak discriminative ability in the diagnostic gray zone (4–10 ng/mL), resulting in a significant number of unnecessary biopsies. PSA density (PSAD), which is computed by normalizing blood PSA to the sonographic prostate volume, may improve cancer diagnostic accuracy. The purpose of this study was to identify appropriate PSAD cutoff values for prostate cancer (PC) diagnosis and to evaluate the diagnostic performance of PSAD obtained using transrectal ultrasound (TRUS) and transabdominal ultrasound (TAUS) volumetry.

**Methods:**

This retrospective, single-center diagnostic accuracy study included 400 men aged 50–80 years with total serum PSA levels between 4 and 10 ng/mL who underwent a standard 12-core TRUS-guided prostate biopsy in the Department of Urology. TRUS and TAUS measured prostate volume separately. PSAD values were derived for each modality and compared with histopathology results using receiver operating characteristic curve analysis. Sensitivity, specificity, positive predictive value, negative predictive value, and likelihood ratios were calculated for four predetermined PSAD thresholds (0.125, 0.150, 0.175, and 0.200 ng/mL/mL).

**Results:**

PC was identified in 171 of 400 men (42.8%). Total PSA alone demonstrated poor discriminatory performance area under the curve (AUC 0.58; 95% CI, 0.53–0.64). TRUS-derived PSAD achieved an AUC of 0.73 (95% CI, 0.67–0.78), with an optimal threshold within this cohort of 0.175 ng/mL/mL yielding sensitivity 69%, specificity 72%, positive likelihood ratio 2.47, and negative likelihood ratio 0.43. TAUS-derived PSAD achieved an AUC of 0.70 (95% CI, 0.65–0.75), with an optimal threshold of 0.150 ng/mL/mL (sensitivity 61% and specificity 72%). The PSAD/free PSA ratio based on TRUS yielded the highest overall AUC (0.74; 95% CI, 0.69–0.79). On multivariable logistic regression adjusting for age, prostate volume, free PSA, and body mass index (BMI), PSAD–TRUS remained an independent predictor of PC (adjusted odds ratio 1.47 per 0.05 ng/mL/mL increase; 95% CI, 1.24–1.75; *P* < 0.001).

**Conclusions:**

PSAD substantially outperforms total PSA for PC detection in men with gray-zone PSA values and remains an independent predictor after adjustment for age, prostate volume, free PSA, and BMI. TRUS-based PSAD at 0.175 ng/mL/mL provides the most clinically balanced diagnostic profile within this cohort, whilst TAUS-based PSAD at 0.150 ng/mL/mL represents a valid and accessible alternative when TRUS is unavailable. These findings support integration of PSAD into biopsy decision algorithms to reduce unnecessary prostatic biopsies, although population-specific validation of these thresholds is required before wider adoption.

## Introduction

With roughly 1.4 million new cases diagnosed each year, prostate cancer (PC) is the second most frequent malignancy in males globally^[^1^]^. It is the most commonly diagnosed non-cutaneous malignancy in developed nations, as well as the third leading cause of cancer-related death in males^[^2,3^]^. In Iran, the epidemiological burden is comparable; PC is the most prevalent non-cutaneous malignancy and the sixth leading cause of cancer mortality among Iranian males, with an age-standardized incidence rate of 21.1 per 100 000^[^4–6^]^. This burden is projected to increase as the population ages and Western living habits become more prevalent, emphasizing the need for more precise and efficient diagnostic procedures^[^7,8^]^.

Prostate-specific antigen (PSA), a serine protease produced by prostatic epithelial cells, is still the most often utilized biomarker for PC diagnosis and monitoring^[^9^]^. However, its clinical value is severely restricted by low specificity, particularly in the so-called gray zone of 4–10 ng/mL, where an increase in PSA might result from benign prostatic hyperplasia (BPH), prostatitis, or cancer^[^10^]^. It is estimated that 30–50% of biopsies performed in response to gray-zone PSA readings are histologically benign, resulting in needless procedural risk, patient concern, and higher healthcare costs^[^10–12^]^. Biopsy-related consequences, including infection, hematuria, and urinary retention, are well established and underscore the need to reduce unnecessary biopsy referrals^[^13^]^.

PSA density (PSAD), which is calculated by dividing the serum PSA concentration by the sonographically determined prostate volume, was added to account for the PSA contribution of benign glandular tissue and therefore enhance the distinction between BPH and PC^[^14,15^]^. PSAD normalizes PSA to prostate size, providing a more tissue-specific signal. Values over 0.15 ng/mL^2^ have been linked to a higher risk of clinically significant malignancy^[^15^]^. PSAD has been incorporated into modern diagnostic algorithms, alongside multiparametric magnetic resonance imaging (mpMRI) and the Prostate Imaging Reporting and Data System, where it aids in biopsy decision-making, particularly in equivocal imaging scenarios^[^16,17^]^.

Despite its conceptual advantages, the clinical application of PSAD is dependent on accurate prostate volume measurement. Transrectal ultrasound (TRUS) is the conventional reference method owing to its anatomical proximity to the gland and established reproducibility; however, transabdominal ultrasound (TAUS) represents a less invasive, more widely accessible alternative that may carry practical advantages in certain clinical settings^[^18^]^. To date, the optimal PSAD threshold varies considerably across published series, ranging from 0.15 to 0.22 ng/mL^2^ in studies from South Korea, Israel, Thailand, China, and Nigeria, reflecting differences in PSA range, population cancer prevalence, measurement methodology, and racial or epidemiological characteristics^[^19–23^]^. Notably, population-specific cutoff data for Middle Eastern men remain limited, and no study to date has directly compared the diagnostic performance of TRUS- and TAUS-derived PSAD in this demographic.

The present study, therefore, aimed to determine the optimal PSAD cutoff value for detecting PC in men aged 50–80 years presenting with PSA levels in the gray zone (4–10 ng/mL) and to evaluate the comparative diagnostic accuracy of PSAD derived from TRUS and TAUS volume measurements. By establishing evidence-based, population-specific thresholds, this study seeks to support more targeted biopsy decision-making and contribute to the reduction of unnecessary prostatic biopsies in clinical practice. The study has also been reported in accordance with the STROCSS criteria^[^24^]^.

## Methods and materials

### Study design and setting

This was a retrospective, single-center study of diagnostic data, which were retrieved from the medical records of male patients who had attended the Urology Department of a tertiary hospital and who had undergone primary TRUS-guided prostate biopsy between the dates specified in the institutional records. The study was conducted from 22 June 2024 to 17 September 2025 in full accordance with the Declaration of Helsinki and applicable national regulations governing retrospective medical record review. The retrospective design allowed the analysis of prospectively collected clinical data without imposing additional procedural demands on participants, while preserving the integrity of successive case accumulation.

HIGHLIGHTS

- Prostate-specific antigen density (PSAD) demonstrated significantly better diagnostic performance than total prostate-specific antigen (PSA) for detecting prostate cancer (PC) in patients with PSA levels between 4 and 10 ng/mL.
- Transrectal ultrasound (TRUS)-derived PSAD achieved the best diagnostic accuracy area under the curve (AUC = 0.73), with an optimal cutoff value of 0.175 ng/mL/mL for predicting PC.
- Transabdominal ultrasound (TAUS)-derived PSAD also showed acceptable discrimination (AUC = 0.70), with an optimal cutoff value of 0.150 ng/mL/mL, providing a practical alternative when TRUS is unavailable.
- The TRUS-based PSAD-to-free PSA ratio showed the highest overall diagnostic performance (AUC = 0.74), suggesting additional value for risk stratification.
- Incorporating PSAD into biopsy decision-making may reduce unnecessary prostate biopsies while maintaining clinically meaningful cancer detection in men with gray-zone PSA values.

### Study population, eligibility criteria, and sampling

The target population comprised men aged 50–80 years presenting with serum total PSA concentrations between 4 and 10 ng/mL on two independent reports who were scheduled for an initial TRUS-guided prostate biopsy at the study center. Patients were enrolled by consecutive sampling, a non-probability sampling method in which all eligible attendees are included in sequence until the required sample size is achieved, thereby minimizing selection bias within the constraints of a single-center retrospective design.

Patients with an active urinary tract infection at the time of assessment were excluded because concurrent infection elevates serum PSA independently of prostatic pathology and would confound PSAD-based discrimination. Men with abnormal findings on digital rectal examination (DRE) were likewise excluded, as clinical suspicion raised by DRE constitutes an independent indication for biopsy that was considered outside the intended clinical application of PSAD-guided decision-making. Prior prostate surgery was an exclusion criterion owing to the structural and volumetric alterations it imposes on ultrasound-based volumetry and background PSA kinetics.

The flow of records from the source dataset to the final analytic cohort, including the number excluded during data verification and the reasons for exclusion, is summarized in Figure 1.

**Figure 1.**
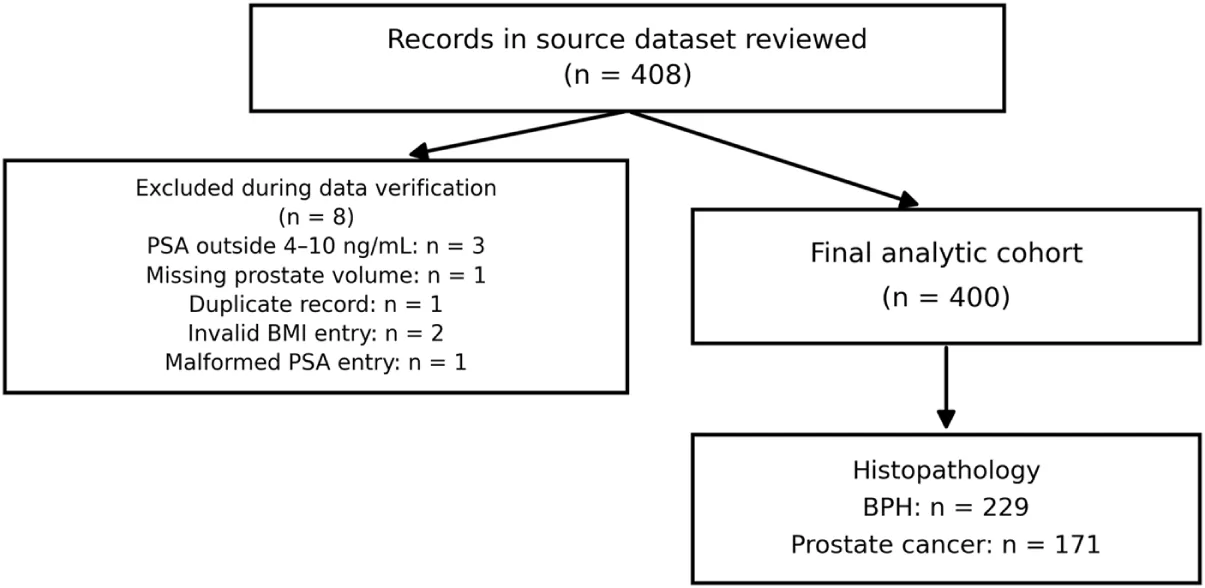
Participant flow and data verification. Of the 408 records in the source dataset, 8 were excluded during data verification (three with total PSA outside the 4–10 ng/mL eligibility range, one with missing prostate volume, one duplicate record, two with invalid BMI entries, and one with a malformed PSA entry), yielding a final analytic cohort of 400 men (171 with PC, 229 with BPH).

Based on values reported in the prior literature, target sensitivity was set at 75% and target specificity at 85%, with a two-sided significance level of α = 0.05 (Zα/2 = 1.96) and statistical power of 80% (Zβ = 0.84). These parameters yielded a minimum requirement of 167 participants (approximately 80 with PC and 87 with BPH). The actual enrolled cohort of 400 patients substantially exceeded this minimum, providing enhanced precision in the estimation of area under the curve (AUC) confidence intervals (CIs) and threshold-specific diagnostic accuracy indices.

The primary outcome (dependent) variable was the result of a 12-core TRUS-guided biopsy, dichotomized as PC (positive) or BPH (negative). Secondary independent variables included age, body mass index (BMI), prostate volume measured by each modality, total and free serum PSA, and all PSAD-derived indices.

### Serum PSA measurement

Venous blood samples were collected from all participants before any urological manipulation (including DRE or prostate ultrasound). Total serum PSA and free PSA concentrations were measured in the clinical biochemistry laboratory of the study center using standard immunoassay methods. Samples from patients with evidence of active urinary tract infection, recent (within 7 days) catheterization, or any prostatic instrumentation were excluded in accordance with the study eligibility criteria to preclude inflammation-driven PSA elevations from distorting PSAD calculations.

### Prostate volume assessment

Prostate volume was independently estimated using two ultrasound approaches for each participant. TRUS was performed using a high-frequency endorectal probe, with the patient in the left lateral decubitus position, following standard bowel preparation. TAUS was performed with the patient supine and the bladder moderately filled. In both modalities, three orthogonal dimensions of the prostate (longitudinal, anteroposterior, and transverse) were obtained, and glandular volume was calculated using the ellipsoid approximation formula: *V* = 0.52 × *L* × *W* × *H*, where *L* is the greatest longitudinal diameter, *W* the transverse diameter, and *H* the anteroposterior diameter, all measured in centimeters.

The inclusion of both TRUS and TAUS volumetry was a pre-specified study objective, allowing a direct within-cohort comparison of the diagnostic performance of PSAD calculated by each method. TRUS-derived volumes are considered the reference standard owing to the proximity of the endorectal probe to the gland; TAUS-derived volumes were evaluated as a potentially more accessible surrogate in resource-limited settings or when patients are intolerant.

### Calculation of PSAD and derived indices

PSAD was calculated by dividing the serum total PSA concentration (ng/mL) by the corresponding sonographically estimated prostate volume (mL), yielding a value expressed as ng/mL/mL. This computation was performed separately for each of the two imaging modalities, producing PSAD–TRUS and PSAD–TAUS as independent study variables. Free PSAD was calculated analogously using the free PSA concentration in the numerator. An additional composite index, the ratio of TRUS-based PSAD to the free-to-total PSA fraction (fPSA/tPSA), was also evaluated as an exploratory parameter.

### Biopsy protocol and histopathological assessment

All biopsy specimens were processed in 10% neutral-buffered formalin and embedded in paraffin. Histopathological evaluation was performed by a single experienced uropathologist who was blinded to the PSAD values at the time of reporting. Each specimen was classified as either BPH or malignant prostate adenocarcinoma. In cases of malignancy, a Gleason score was assigned, and the corresponding ISUP Grade Group was determined according to the 2014 International Society of Urological Pathology consensus classification^[^25^]^.

The 12-core protocol was adopted as the reference standard biopsy strategy in accordance with contemporary urological guidelines. It is acknowledged that this technique samples approximately 1% of the total glandular volume and carries an inherent false-negative rate, particularly for anteriorly situated tumors; this represents a recognized limitation of the study design and is discussed in the limitations subsection.

### Statistical analysis

All statistical analyses were performed using SPSS Statistics version 25.0 (IBM Corporation, Armonk, NY, USA). Continuous variables are reported as mean ± standard deviation, and categorical variables as absolute frequencies and percentages. The normality of distributions of continuous variables was evaluated prior to group comparisons using the Kolmogorov–Smirnov test.

Between-group differences in continuous variables (BPH vs. PC) were assessed using the independent-samples Student’s *t*-test, as data distributions satisfied the assumptions of approximate normality and homogeneity of variances. A two-tailed significance level of *P* < 0.05 was adopted throughout.

The discriminatory capacity of each PSA-derived index and volumetric parameter was evaluated by constructing receiver operating characteristic (ROC) curves, with histopathological biopsy outcome as the reference standard. The area under the ROC curve (AUC) was estimated for each parameter, together with its 95% CI, computed by the DeLong non-parametric method. AUC values were interpreted according to conventional benchmarks: values between 0.70 and 0.79 indicate acceptable-to-moderate discrimination, values between 0.80 and 0.89 indicate good discrimination, and values ≥ 0.90 indicate excellent discrimination.

For PSAD specifically, sensitivity, specificity, positive predictive value (PPV), negative predictive value (NPV), and likelihood ratios (positive, LR+; negative, LR−) were computed at four pre-specified threshold values (0.125, 0.150, 0.175, and 0.200 ng/mL/mL) for both TRUS- and TAUS-derived volumes. Ninety-five per cent CIs for LR+ and LR− were derived from the corresponding sensitivity, specificity, and group sample sizes using standard log-transformation methods. The optimal threshold within this cohort for each imaging modality was defined as the cut-off yielding the most favorable and clinically balanced combination of sensitivity and specificity, assessed with reference to the Youden index (*J* = sensitivity + specificity − 1). As this threshold was derived empirically from the study sample, it should be regarded as cohort-specific pending external validation. Ninety-five percent CIs for sensitivity and specificity were calculated using the Wilson score interval method.

To determine whether PSAD–TRUS was independently associated with PC after accounting for other clinically relevant variables, a multivariable binary logistic regression model was constructed with histopathological outcome (PC vs BPH) as the dependent variable and PSAD–TRUS, age, TRUS-derived prostate volume, free PSA, and BMI as covariates. Adjusted odds ratios (aORs) with 95% CIs were derived from the model coefficients and their standard errors, and model fit was summarized using the likelihood-ratio chi-square test and Nagelkerke and McFadden pseudo-R^2^.

No imputation was performed for missing data; participants with incomplete records were excluded from the final analysis, yielding a final analytic cohort of *n* = 400, used for both univariable and multivariable analyses. Given that the enrolled cohort (*n* = 400) substantially exceeded the pre-calculated minimum sample size, there was no material concern regarding statistical power for the primary ROC comparisons.

## Results

### Study population and baseline characteristics

A total of 400 men aged 50–80 years with serum total PSA levels between 4 and 10 ng/mL who underwent 12-core TRUS-guided prostate biopsy were included in the final analysis. Histopathological examination identified PC in 171 patients (42.8%) and BPH in 229 patients (57.3%).

The mean age of the overall cohort was 67.94 ± 6.95 years. On an independent-samples *t*-test, age was significantly higher in the PC group (69.44 ± 7.21 years) than in the BPH group (66.82 ± 6.55 years; *P* < 0.001); however, these figures are not clinically meaningful and are provided for reporting. By contrast, body weight, height, and BMI did not differ significantly between groups (*P* = 0.509, 0.682, and 0.334, respectively), indicating that anthropometric factors other than age were broadly comparable between the two diagnostic strata (Table 1).

**Table 1:** Baseline demographic characteristics by diagnostic group (BPH vs. prostate cancer).

Variable	BPH mean	BPH SD	PC mean	PC SD	*P* value
Age (years)	66.82	6.55	69.44	7.21	<0.001
Weight (kg)	75.92	10.71	76.70	12.68	0.509
Height (m)	1.71	0.06	1.70	0.07	0.682
BMI (kg/m^2^)	25.92	3.21	26.27	3.85	0.334

BPH, benign prostatic hyperplasia; PC, prostate cancer; BMI, body mass index; SD, standard deviation. *P*-values derived from an independent-samples *t*-test.

### Prostate volume, PSA parameters, and PSAD indices

Prostate volume estimated by both TRUS and TAUS was significantly greater in the BPH group than in the PC group (TRUS: 53.33 ± 24.61 mL vs. 38.34 ± 21.42 mL, *P* < 0.001; TAUS: 62.58 ± 28.35 mL vs. 47.87 ± 23.80 mL, *P* < 0.001). Residual urine volume did not differ significantly between the groups (40.09 ± 53.12 mL vs. 42.13 ± 61.61 mL, *P* = 0.722). Free PSA was likewise higher in the BPH group (1.48 ± 0.84 ng/mL vs. 1.24 ± 0.79 ng/mL, *P* = 0.005).

Conversely, mean serum total PSA was significantly higher in the PC group (7.65 ± 1.72 ng/mL vs. 7.10 ± 1.80 ng/mL; *P* = 0.002), as was PSAD calculated by both TRUS-derived volume (0.25 ± 0.13 vs. 0.16 ± 0.08 ng/mL/mL; *P* < 0.001) and TAUS-derived volume (0.19 ± 0.09 vs. 0.13 ± 0.06 ng/mL/mL; *P* < 0.001). Free PSAD values were also significantly higher in the PC group for both imaging modalities (TRUS-based free PSAD: 0.04 ± 0.03 vs. 0.03 ± 0.02; *P* = 0.002; TAUS-based free PSAD: 0.03 ± 0.02 vs. 0.02 ± 0.02; *P* = 0.015). These findings confirm that while absolute PSA concentrations differ only modestly within the gray zone, PSA-derived indices that incorporate glandular volume provide substantially greater discriminatory separation between BPH and malignancy (Table 2).

**Table 2:** Comparison of prostate volume and PSA-derived indices between diagnostic groups.

Variable	BPH mean	BPH SD	PC mean	PC SD	*P* value
Prostate volume – TRUS (mL)	53.33	24.61	38.34	21.42	<0.001
Prostate volume – TAUS (mL)	62.58	28.35	47.87	23.80	<0.001
Residual urine volume (mL)	40.09	53.12	42.13	61.61	0.722
Free PSA (ng/mL)	1.48	0.84	1.24	0.79	0.005
Total PSA (ng/mL)	7.10	1.80	7.65	1.72	0.002
PSAD–TRUS (ng/mL/mL)	0.16	0.08	0.25	0.13	<0.001
Free PSAD–TRUS	0.03	0.02	0.04	0.03	0.002
PSAD–TAUS (ng/mL/mL)	0.13	0.06	0.19	0.09	<0.001
Free PSAD–TAUS	0.02	0.02	0.03	0.02	0.015

TRUS, transrectal ultrasound; TAUS, transabdominal ultrasound; PSAD, prostate-specific antigen density; Free PSAD, free PSA density. All comparisons by an independent-samples *t*-test.

### Histopathological grading in the PC group

Among the 171 men with confirmed PC, the distribution of Gleason scores and corresponding ISUP Grade Groups is presented in Table 3. Grade Group 1 (Gleason score ≤6, pattern 3 + 3) accounted for 63 cases (36.8%), and Grade Group 2 (pattern 3 + 4) for 65 cases (38.0%), together comprising the majority of malignancies (74.8%). Grade Group 3 (pattern 4 + 3) was identified in 13 cases (7.6%), while Grade Groups 4 and 5 (Gleason scores 8–10) collectively accounted for 30 cases (17.5%). The predominance of low-to-intermediate-risk histology is consistent with the enrolled PSA range and the use of an initial biopsy strategy.

**Table 3:** Gleason score and ISUP Grade Group distribution in the prostate cancer cohort (*n* = 171).

Gleason score	ISUP grade	Grade group	*n*	% (of PC, *n* = 171)
3 + 3	1	Grade Group 1	63	36.8
3 + 4	2	Grade Group 2	65	38.0
4 + 3	3	Grade Group 3	13	7.6
4 + 4	4	Grade Group 4	8	4.7
3 + 5	4	Grade Group 4	1	0.6
4 + 5	5	Grade Group 5	7	4.1
5 + 3	5	Grade Group 5	2	1.2
5 + 4	5	Grade Group 5	11	6.4
5 + 5	5	Grade Group 5	1	0.6
Total	–	–	171	100.0

ISUP, International Society of Urological Pathology (2014 classification). Percentages are of the prostate cancer group (*n* = 171).

Subgroup analyses comparing men with Gleason score 3 + 3 against the BPH group, and then against the remaining PC cases, revealed no statistically significant between-group differences for any prostate volume or PSA-index variable (all *P* > 0.05). This observation suggests that PSAD-based discrimination at the lower end of the malignancy spectrum may be less pronounced and merits consideration when applying threshold-based decision criteria in clinical practice.

### Receiver operating characteristic curve analysis

ROC curve analysis was performed to evaluate the discriminatory capacity of each PSA-derived index and volumetric parameter for the detection of PC. Results are summarized in Table 4.

**Table 4:** ROC curve analysis: discriminatory performance of prostate volume and PSA-derived indices for prostate cancer detection.

Parameter	AUC	95% CI lower	95% CI upper	*P* value
PSAD–TRUS (ng/mL/mL)	0.73	0.67	0.78	<0.001
PSAD/fPSA ratio – TRUS	0.74	0.69	0.79	<0.001
PSAD–TAUS (ng/mL/mL)	0.70	0.65	0.75	<0.001
Prostate volume – TRUS (mL)	0.70	0.65	0.75	<0.001
Prostate volume – TAUS (mL)	0.67	0.62	0.73	<0.001
Total PSA (ng/mL)	0.58	0.53	0.64	0.003
Free PSAD – TRUS	0.58	0.51	0.63	0.009
Free PSAD – TAUS	0.55	0.48	0.60	0.110

AUC, area under the receiver operating characteristic curve; 95% CI, 95% confidence interval; TRUS, transrectal ultrasound; TAUS, transabdominal ultrasound; PSAD, PSA density. AUC values >0.70 are highlighted in the main text as indicators of moderate-to-good discrimination.

Total serum PSA alone demonstrated limited discriminatory capacity within the gray zone, yielding an AUC of only 0.58 (95% CI: 0.53–0.64; *P* = 0.003). In contrast, PSAD calculated from TRUS-derived volume achieved an AUC of 0.73 (95% CI: 0.67–0.78; *P* < 0.001), and PSAD from TAUS-derived volume achieved an AUC of 0.70 (95% CI: 0.65–0.75; *P* < 0.001). The composite index representing the ratio of TRUS-based PSAD to the free-to-total PSA fraction yielded the highest AUC in the present study at 0.74 (95% CI: 0.69–0.79; *P* < 0.001), suggesting that combining density-adjusted PSA with the free PSA fraction confers additive diagnostic information.

Prostate volume alone demonstrated moderate discriminatory ability, with AUCs of 0.70 (TRUS) and 0.67 (TAUS), consistent with the well-established inverse relationship between glandular volume and cancer detection rate. Free PSAD based on TRUS provided marginal discrimination (AUC, 0.58; *P* = 0.009), whereas free PSAD based on TAUS did not reach statistical significance (AUC, 0.55; 95% CI, 0.48–0.60; *P* = 0.110) and therefore should not be used as an independent discriminator in this patient population.

In an exploratory supplementary analysis, patients with Gleason score 3 + 3 (ISUP Grade Group 1), who are generally considered low-risk candidates for active surveillance, were temporarily reclassified into the BPH group. This approach was undertaken to investigate whether excluding these low-risk cancers from the malignant category would improve the discriminatory performance of PSAD-based diagnostic models. Contrary to expectations, ROC analysis demonstrated no improvement in diagnostic performance; instead, AUC values decreased after reclassification.

### Multivariable analysis

To determine whether PSAD–TRUS was independently associated with PC after accounting for other clinically relevant variables, a multivariable binary logistic regression model was constructed with age, TRUS-derived prostate volume, free PSA, and BMI as covariates (*n* = 400). The overall model was statistically significant (likelihood-ratio χ^2^ = 98.0, df = 5, *P* < 0.001), with a Nagelkerke pseudo-*R*^2^ of 0.24 (McFadden pseudo-*R*^2^ 0.18), indicating moderate explanatory power.

PSAD–TRUS was a strong, statistically independent predictor of PC after adjustment, with an aOR of 1.47 per 0.05 ng/mL/mL increase (95% CI: 1.24–1.75; *P* < 0.001). Age was also independently associated with PC(aOR 1.09 per year; 95% CI: 1.05–1.13; *P* < 0.001), as was BMI (aOR 1.09 per kg/m^2^; 95% CI: 1.02–1.16; *P* = 0.011). Free PSA was inversely associated with PC after adjustment (aOR 0.61 per ng/mL; 95% CI: 0.44–0.84; *P* = 0.003). TRUS-derived prostate volume was not independently associated with the outcome once PSAD–TRUS was included in the model (aOR 0.95 per 10 mL; 95% CI: 0.81–1.12; *P* = 0.564), consistent with prostate volume’s contribution being captured within the PSAD index itself. Full model results are presented in Table 5.

**Table 5:** Multivariable logistic regression for prostate cancer, adjusted for age, TRUS-derived prostate volume, free PSA, and body mass index (*n* = 400).

Variable	aOR	95% CI lower	95% CI upper	*P* value
PSAD–TRUS (per 0.05 ng/mL/mL)	1.47	1.24	1.75	<0.001
Age (per year)	1.09	1.05	1.13	<0.001
Prostate volume – TRUS (per 10 mL)	0.95	0.81	1.12	0.564
Free PSA (per ng/mL)	0.61	0.44	0.84	0.003
BMI (per kg/m^2^)	1.09	1.02	1.16	0.011

aOR, adjusted odds ratio; CI, confidence interval; BMI, body mass index; PSAD, prostate-specific antigen density. Model: likelihood-ratio χ^2^ = 98.0, df = 5, *P* < 0.001; Nagelkerke pseudo-*R*^2^ = 0.24.

### Diagnostic performance at specific PSAD thresholds

To identify operationally useful cut-off values, sensitivity, specificity, PPV, NPV, and likelihood ratios (LR+ and LR−), with 95% CIs, were calculated for PSAD at four pre-specified thresholds (0.125, 0.150, 0.175, and 0.200 ng/mL/mL) for each imaging modality. Complete diagnostic accuracy indices are presented in Table 6.

**Table 6:** Diagnostic accuracy indices for PSAD at four threshold levels using TAUS- and TRUS-derived prostate volumes.

Method	Threshold	Sens.	Spec.	PPV	NPV	LR+ (95% CI)	LR− (95% CI)	Note
TAUS	≥0.125	74%	55%	55%	74%	1.67 (1.40–1.95)	0.46 (0.35–0.62)	
TAUS	≥0.150	61%	72%	62%	71%	2.20 (1.71–2.77)	0.54 (0.44–0.67)	Optimal*
TAUS	≥0.175	51%	77%	62%	68%	2.22 (1.66–2.90)	0.63 (0.54–0.76)	
TAUS	≥0.200	41%	86%	69%	66%	3.02 (2.03–4.23)	0.68 (0.60–0.79)	
TRUS	≥0.125	84%	39%	51%	77%	1.38 (1.22–1.56)	0.41 (0.28–0.60)	
TRUS	≥0.150	76%	57%	57%	76%	1.76 (1.50–2.11)	0.42 (0.31–0.56)	
TRUS	≥0.175	69%	72%	65%	76%	2.47 (1.96–3.11)	0.43 (0.34–0.55)	Optimal*
TRUS	≥0.200	62%	76%	66%	73%	2.63 (1.99–3.34)	0.50 (0.41–0.61)	

Sens., sensitivity; Spec., specificity; PPV, positive predictive value; NPV, negative predictive value; LR+, positive likelihood ratio; LR−, negative likelihood ratio; TAUS, transabdominal ultrasound; TRUS, transrectal ultrasound.

^*^ The cut-off identified as optimal within this cohort for each modality, selected on the basis of the most favorable balance between sensitivity and specificity; population-specific validation is required before generalization. All values represent point estimates derived from the 400-patient cohort; 95% CIs for sensitivity and specificity are reported in the main text, and 95% CIs for LR+ and LR− are reported in Table 6.

For TAUS-based PSAD, a clear trade-off between sensitivity and specificity was observed across the threshold range. At a cut-off of 0.125 ng/mL/mL, sensitivity reached 74%, but specificity fell to 55% [LR+ 1.67 (95% CI: 1.40–1.95), LR− 0.46 (95% CI: 0.35–0.62)], indicating a high false-positive burden. At the opposite extreme, a threshold of 0.200 ng/mL/mL yielded the highest specificity in the TAUS series (86%), albeit with a markedly reduced sensitivity of 41% [LR+ 3.02 (95% CI: 2.03–4.23), LR− 0.68 (95% CI: 0.60–0.79)], potentially allowing a clinically relevant proportion of cancers to be missed. A threshold of 0.150 ng/mL/mL provided the most balanced performance for TAUS-based PSAD, with sensitivity 61% (95% CI: 54–69%), specificity 72% (95% CI: 66–78%), PPV 62%, NPV 71%, and LR+ 2.20 (95% CI: 1.71–2.77). This threshold was therefore identified as the optimal cut-off for TAUS-based PSAD in the present cohort.

For TRUS-based PSAD, the analogous pattern was observed. The lowest threshold evaluated (0.125 ng/mL/mL) yielded high sensitivity (84%) but poor specificity (39%), rendering it unsuitable for reducing unnecessary biopsies. At a threshold of 0.200 ng/mL/mL, specificity increased to 76%, but sensitivity fell to 62%. A threshold of 0.175 ng/mL/mL achieved the most favorable balance for TRUS-based PSAD, with sensitivity 69% (95% CI: 62–76%), specificity 72% (95% CI: 66–78%), PPV 65%, NPV 76%, LR+ 2.47 (95% CI: 1.96–3.11), and LR− 0.43 (95% CI: 0.34–0.55). This combination of sensitivity and specificity represents the optimal operating point within this cohort for TRUS-based PSAD in this population; as with the TAUS-based threshold, external validation in an independent population is required before this cut-off is adopted more widely.

Across all thresholds examined, TRUS-based PSAD consistently provided higher sensitivity at equivalent levels of specificity compared with TAUS-based PSAD, reflecting the greater precision of endorectal volumetry. Nevertheless, the TAUS-based index achieved clinically acceptable discrimination (AUC 0.70; at the optimal threshold in this cohort, sensitivity/specificity of 61 and 72%), supporting its use as a practical alternative when TRUS is unavailable or not tolerated.

## Discussion

This study evaluated the diagnostic performance of PSAD derived from TRUS and TAUS in 400 men aged 50–80 years presenting with grey-zone PSA levels (4–10 ng/mL). In men with “gray zone PSA levels (4–10 ng/mL). In this cohort, PSAD is a highly accurate predictor of PC. According to studies conducted on the Iranian population, PSAD enhances diagnostic specificity greatly, reducing the need for unnecessary biopsies when compared to total PSA alone^[^26^]^. The central finding is that both PSAD indices substantially outperformed total PSA in discriminating PC from BPH, with total PSA yielding an AUC of only 0.58 compared with 0.73 and 0.70 for TRUS-based and TAUS-based PSAD, respectively. TRUS-based PSAD demonstrated the strongest overall diagnostic utility, with an optimal threshold within this cohort of 0.175 ng/mL^2^. TAUS-based PSAD, at an optimal threshold within this cohort of 0.150 ng/mL^2^, performed only marginally below its TRUS counterpart, supporting its use as a viable alternative in settings where TRUS is unavailable or poorly tolerated. On multivariable logistic regression, PSAD–TRUS remained independently associated with PC after adjustment for age, prostate volume, free PSA, and BMI (adjusted OR 1.47 per 0.05 ng/mL/mL increase; 95% CI 1.24–1.75; *P* < 0.001), indicating that its diagnostic value is not simply attributable to its correlation with these other clinical variables. Taken together, these findings indicate that incorporating PSAD into the biopsy decision pathway may meaningfully improve risk stratification for men with gray zone PSA and that clinically acceptable diagnostic performance can be achieved with either ultrasound modality.

The limited diagnostic value of total PSA within the gray zone is well established, and the present findings are consistent with this evidence. Total PSA yielded an AUC of only 0.58 in this cohort, a figure that is statistically indistinguishable from chance and corroborates the widely recognized problem of PSA’s poor PPV in this range^[^10,27^]^. In contrast, TRUS-based PSAD achieved an AUC of 0.73 and TAUS-based PSAD an AUC of 0.70, improvements of 0.15 and 0.12 AUC units, respectively, over total PSA alone. This pattern of PSAD superiority has been consistently observed across multiple international cohorts; a Chinese study by Nan *et al* reported a total PSA AUC of 0.62 against a PSAD–TRUS AUC of 0.83^[^28^]^, while a 2024 Chinese study by Wang *et al* documented an even more pronounced disparity (total PSA AUC 0.36 vs. PSAD–TRUS AUC 0.70)^[^15^]^. A Slovak study published in the same year recorded AUC values of 0.63 and 0.71 for total PSA and PSAD–TRUS, respectively^[^29^]^, and Zhang *et al* in China reported corresponding values of 0.59 and 0.77^[^30^]^. The present results align with this literature, reinforcing the case that PSAD, rather than total PSA in isolation, should be integrated into the biopsy decision pathway for men in the gray zone.

This study found that the best TRUS-based PSAD threshold within this cohort was 0.175 ng/mL^2^, with a sensitivity of 69%, specificity of 72%, positive likelihood ratio (LR+) of 2.47 (95% CI, 1.96–3.11), and negative likelihood ratio (LR−) of 0.43 (95% CI, 0.34–0.55). These likelihood ratio values show that a positive result significantly increases the post-test probability of malignancy, but a negative result offers considerable reassurance against cancer in this PSA range. The identified threshold of 0.175 ng/mL^2^ falls within the range of 0.15–0.20 ng/mL^2^ commonly reported in the international literature. Park and Yu, in South Korea, found an optimal PSAD–TRUS threshold of 0.218 ng/mL^2^ (sensitivity 59.5%, specificity 84.8%) in men with PSA of 2.5–20 ng/mL^[^21^]^; Yusim *et al*, in Israel, reported a threshold of 0.15 ng/mL^2^ (sensitivity and specificity both approximately 70%)^[^31^]^; and a Thai cohort study determined 0.20 ng/mL^2^ as optimal (sensitivity 67.3%, specificity 62.1%)^[^20^]^. Multiple Chinese investigations have similarly converged on thresholds of 0.15–0.16 ng/mL^2^ in the PSA 4–10 ng/mL range^[^32,33^]^. The modest variation across studies is attributable to differences in PSA range, population prevalence of PC, racial and epidemiological characteristics, and operator-dependent variation in volume estimation, all factors acknowledged in this study^[^34^]^. The present thresholds are therefore consistent with, rather than discrepant from, the established body of evidence.

A clinically important finding of this study is the acceptable diagnostic performance of TAUS-based PSAD (AUC = 0.70), which was only marginally inferior to its TRUS-based counterpart. The optimal TAUS threshold within this cohort was 0.150 ng/mL^2^ (sensitivity 61% and specificity 72%), values comparable to those reported in a Nigerian cohort study where TAUS-PSAD at 0.15 ng/mL^2^ achieved a sensitivity of 69.8% and a specificity of 89.5%^[^22^]^. The recent Chinese study by Xu *et al*, employing TAUS-based volume measurements, reported an even higher AUC of 0.81^[^35^]^, suggesting that transabdominal volume estimation may carry greater precision in certain clinical or operator-proficiency contexts. Although TRUS remains the methodological gold standard for prostate volume measurement owing to its superior anatomical resolution, the present data indicate that TAUS-derived PSAD is a valid and accessible alternative^[^36^]^. In resource-limited settings or where TRUS is unavailable, poorly tolerated, or contraindicated, clinicians may reasonably employ TAUS-based PSAD as an adjunct to clinical assessment without substantially compromising diagnostic accuracy.

The AUC values for PSAD in this study (0.70–0.73) are contextually comparable to the international literature. AUCs for PSAD-TRUS range from 0.63 in a 2021 Chinese consortium study^[^33^]^ to 0.78 in an Israeli series^[^31^]^, with intermediate values of around 0.66^[^32^]^, 0.69 in Thailand^[^20^]^, 0.70 in a 2015 Chinese multicenter trial^[^37^]^, and 0.76 in South Korea^[^21^]^. The present study’s AUC of 0.73 falls within this well-defined range, providing external validity to the findings. Minor variations across populations are likely due to the interaction of different cancer prevalences, biopsy techniques, ultrasound techniques, and patient selection criteria, rather than underlying variation in the biological relationship between prostate volume and cancer risk^[^38,39^]^.

## Limitations

Several limitations in this study should be considered. First, the retrospective, single-center approach limits external generalizability because the study population’s epidemiological and demographic features may differ from those of other centers or foreign populations. Second, prostate volume was measured by multiple operators using two ultrasound modalities; given the inherent operator-dependent nature of ultrasonographic volume estimation, inter- and intra-observer variability cannot be ruled out, and measurement error may have been introduced into both PSAD indices. Third, this study was limited to men with PSA levels in the 4–10 ng/mL gray zone, and the optimal thresholds discovered here cannot be generalized to populations with PSA levels outside of this range. Fourth, advanced imaging modalities such as mpMRI and contemporary biomarkers were not included because it was not feasible to use them owing to sample size and cost constraints for patients and the study, preventing direct comparison of PSAD to these new diagnostic tools. Fifth, the multivariable model was restricted to four covariates (age, prostate volume, free PSA, and BMI) selected on clinical grounds; because PSAD is itself derived mathematically from PSA and prostate volume, some collinearity between these terms is expected, and the model should be interpreted as evidence that PSAD retains explanatory value beyond these individual components rather than as a fully independent causal estimate. Future research should address these limitations using multicenter prospective designs, standardized volume measurement techniques, and head-to-head comparisons with mpMRI-based diagnostic algorithms.

## Conclusion

The present study demonstrates that PSAD substantially improves discriminatory performance over total PSA alone in men presenting with gray-zone PSA levels, and this association persists after adjustment for age, prostate volume, free PSA, and BMI on multivariable analysis. TRUS-based PSAD, with an optimal threshold in this cohort of 0.175 ng/mL^2^, provides the most clinically balanced sensitivity–specificity profile and should be considered a first-line adjunct to total PSA in biopsy decision-making within this range, subject to external validation. TAUS-based PSAD, with a threshold in this cohort of 0.150 ng/mL^2^ represents a valid and accessible alternative when TRUS is unavailable. These findings support further evaluation of the integration of PSAD, irrespective of measurement modality, into risk-stratification algorithms for gray-zone PSA, with the potential to reduce unnecessary biopsies and associated morbidity without clinically significant loss of cancer detection.

## Data Availability

The datasets generated and/or analyzed during the current study are not publicly available due to institutional privacy policies, but are available from the corresponding author on reasonable request.

## References

[1] Bergengren O Pekala KR Matsoukas K. 2022 update on prostate cancer epidemiology and risk factors-a systematic review. Eur Urol 2023;84:191–206. 10.1016/j.eururo.2023.04.021 PMC10851915 37202314

[2] Kensler KH Rebbeck TR. Cancer progress and priorities: prostate cancer. Cancer Epidemiol Biomarkers Prev 2020;29:267–77. 10.1158/1055-9965.EPI-19-0412 PMC7006991 32024765

[3] Desai MM Cacciamani GE Gill K. Trends in incidence of metastatic prostate cancer in the US. JAMA Netw Open 2022;5:e222246e. 10.1001/jamanetworkopen.2022.2246 PMC9907338 35285916

[4] Nowroozi M Mosa Farkhani E Hushmandi K. Trends in incidence of prostate cancer in Iran and Its 31 provinces during 2003 - 2016. Int J Cancer Manag 2023;16:e136819.

[5] Zendehdel K. Cancer Statistics in I.R. Iran in 2020. Basic & Clinical Cancer Research 2021;12:159–65.

[6] Hassanipour S Delam H Arab-Zozani M. Survival rate of prostate cancer in asian countries: a systematic review and meta-analysis. Ann Glob Health 2020;86:2. 10.5334/aogh.2607 PMC6966336 31976303

[7] Askarpour H Hassan Lotfi M Fallahzadeh H. Risk factors for prostate cancer in Kohgiluyeh and BoyerAhmad provinces, Iran. Indian J Forensic Med Toxicol 2021;15:2464–72.

[8] Momayyezi M Dehghani Tafti A Keyghobadi N. Prevalence of prostate cancer and its risk factors: results of the first phase of shahedieh cohort study in Yazd Province. Iran Hums-jpm 2022;9:218.

[9] Raychaudhuri R Lin DW Montgomery RB. Prostate cancer: a review. Jama 2025;333:1433–46. 10.1001/jama.2025.0228 40063046

[10] Merriel SWD Pocock L Gilbert E. Systematic review and meta-analysis of the diagnostic accuracy of prostate-specific antigen (PSA) for the detection of prostate cancer in symptomatic patients. BMC Med 2022;20:54. 10.1186/s12916-021-02230-y PMC8819971 35125113

[11] Caruana M Gulati R Etzioni R. Benefits and harms of prostate specific antigen testing according to Australian guidelines. Int J Cancer 2024;154:648–58. 10.1002/ijc.34731 PMC11296206 37819139

[12] Lophatananon A Light A Burns-Cox N. Re-evaluating the diagnostic efficacy of PSA as a referral test to detect clinically significant prostate cancer in contemporary MRI-based image-guided biopsy pathways. J Clin Urol 2023;16:264–73. 10.1177/20514158211059057 PMC7614972 37614642

[13] Mian BM Feustel PJ Aziz A. Complications following transrectal and transperineal prostate biopsy: results of the ProBE-PC randomized clinical trial. J Urol 2024;211:205–13. 10.1097/JU.0000000000003788 37976319

[14] Wang F Fu M Tang Y. The value of adjusted PSAD in prostate cancer detection in the Chinese population. Front Oncol 2024;14:1462997. 10.3389/fonc.2024.1462997 PMC11479861 39416462

[15] Chou Y-J Jong B-E Tsai Y-C. Integrating PSA change with PSA density enhances diagnostic accuracy and helps avoid unnecessary prostate biopsies. Diagnostics 2025;15:2027. 10.3390/diagnostics15162027 PMC12385582 40870878

[16] Haj-Mirzaian A Burk KS Lacson R. Magnetic resonance imaging, clinical, and biopsy findings in suspected prostate cancer: a systematic review and meta-analysis. JAMA Netw Open 2024;7:e244258e. 10.1001/jamanetworkopen.2024.4258 PMC10980971 38551559

[17] Wang S Kozarek J Russell R. Diagnostic performance of prostate-specific antigen density for detecting clinically significant prostate cancer in the era of magnetic resonance imaging: a systematic review and meta-analysis. Eur Urol Oncol 2024;7:189–203. 10.1016/j.euo.2023.08.002 PMC12826839 37640584

[18] Leslie SW Carter KR. Transrectal ultrasonography and image-guided biopsies of the prostate. In: StatPearls. StatPearls Publishing LLC.; StatPearls Publishing Copyright © 2026. 2026. 34033331

[19] Haroon H Bashir Q Haroon A. Cut-off values of prostate specific antigen density– an effective screening marker before prostate biopsy. Khyber Med Univ J 2022;14:16–20.

[20] Rhunsiri P Sirisopana K Jenjitranant P. Prediction of a novel prostate-specific antigen density cutoff value and use of transrectal ultrasound-guided prostate biopsy for diagnosis of prostate cancer in Thailand. Insight Urol 2022;43:1–5.

[21] Park DH Yu JH. Prostate-specific antigen density as the best predictor of low- to intermediate-risk prostate cancer: a cohort study. Transl Cancer Res 2023;12:502–14. 10.21037/tcr-22-1855 PMC10080306 37033352

[22] Agbo C Ramyil V Dakum N. Usefulness–of–prostate–specific–antigen–density–in–prostate–cancer–detection–among–men–with–psa–of–410–ngml. Ann Med Health Sci Res 2020;10:1138–41.

[23] Duvedi P Singh H Bedi G. Role of Prostate-Specific Antigen (PSA) in patients with benign prostate hyperplasia. J Clinical DIAGNOSTIC Research 2019;13:BC01–BC03.

[24] Agha RA Mathew G Rashid R. Revised Strengthening the reporting of cohort, cross-sectional and case-control studies in surgery (STROCSS) guideline: an update for the age of Artificial Intelligence. Premier J Science 2025;10:100081.

[25] Epstein JI Egevad L Amin MB. The 2014 International Society of Urological Pathology (ISUP) consensus conference on gleason grading of prostatic carcinoma: definition of grading patterns and proposal for a new grading system. Am J Surg Pathol 2016;40:244–52. 10.1097/PAS.0000000000000530 26492179

[26] Nowroozi M Ayati M Jamshidian H. Transition zone prostate specific antigen density improves prostate cancer detection in Iranian men. Nephrourol Mon 2015;7:e26752. 10.5812/numonthly.26752 PMC4360524 25821749

[27] David MK Leslie SW. Prostate-Specific Antigen. In: StatPearls. StatPearls Publishing LLC.; StatPearls Publishing Copyright © 2026. 2026. 32491427

[28] Nan LB Yin XT Gao JP. Significant Diagnostic Value of Free-Serum PSA (FPSA)/Prostate-Specific Antigen Density (PSAD) and (F/T)/PSAD for Prostate Cancer of the Chinese Population in a Single Institution. Med Sci Monit 2019;25:8345–51. 10.12659/MSM.916900 PMC6859934 31691648

[29] Hrubá T Kubas V Franko M. Precision in prostate cancer detection: integrating prostate-specific antigen density (PSAD) and the Prostate Imaging Reporting and Data System (PI-RADS) to provide additional risk stratification for a more accurate diagnostic decision. Ir J Med Sci 2024;193:2635–42. 10.1007/s11845-024-03771-w PMC11666638 39093531

[30] Zhang Q Li H Song Z. Potential diagnostic value of multiple indicators combined with total prostate-specific antigen in prostate cancer. J Int Med Res 2023;51:3000605231204429. 10.1177/03000605231204429 PMC10586000 37848343

[31] Yusim I Krenawi M Mazor E. The use of prostate specific antigen density to predict clinically significant prostate cancer. Sci Rep 2020;10:20015. 10.1038/s41598-020-76786-9 PMC7672084 33203873

[32] Liu M Hou HM Li X. Value of prostate volume, PSAD and F/T in the detection of prostate cancer in males with PSA 4-10 μg/L. Zhonghua Zhong Liu Za Zhi 2016;38:526–29. 10.3760/cma.j.issn.0253-3766.2016.07.009 27531267

[33] Song ZJ Qian JK Yang Y. PSA density in the diagnosis of prostate cancer in the Chinese population: results from the Chinese prostate cancer consortium. Asian J Androl 2021;23:300–05. 10.4103/aja.aja_61_20 PMC8152427 33208562

[34] Quinn L Tryposkiadis K Deeks J. Interobserver variability studies in diagnostic imaging: a methodological systematic review. Br J Radiol 2023;96:20220972. 10.1259/bjr.20220972 PMC10392644 37399082

[35] Xu Z Song H Huang H. Diagnostic value of transabdominal ultrasound-based versus magnetic resonance imaging-based prostate-specific antigen density for clinically significant prostate cancer. Eur Urol Open Sci 2026;84:70–76. 10.1016/j.euros.2026.01.003 PMC12861266 41631039

[36] Heidenreich A Aus G Bolla M. EAU guidelines on prostate cancer. Eur Urol 2008;53:68–80. 10.1016/j.eururo.2007.09.002 17920184

[37] Lin YR Wei XH Uhlman M. PSA density improves the rate of prostate cancer detection in Chinese men with a PSA between 2.5-10.0 ng ml (−1) and 10.1-20.0 ng ml (−1): a multicenter study. Asian J Androl 2015;17:503–07. 10.4103/1008-682X.142129 PMC4430959 25475661

[38] Wei JT Barocas D Carlsson S. Early detection of prostate cancer: AUA/SUO guideline Part I: prostate cancer screening. J Urol 2023;210:46–53. 10.1097/JU.0000000000003491 PMC11060750 37096582

[39] Pasecinic V Novacescu D Zara F. Predictors of ISUP grade group discrepancies between biopsy and radical prostatectomy: a single-center analysis of clinical, imaging, and histopathological parameters. Cancers (Basel) 2025;17:2595. 10.3390/cancers17152595 PMC12346174 40805290

